# Phenotypic comparison of idiopathic epilepsy in English Springer Spaniels and Cocker Spaniels using a validated owner-completed questionnaire

**DOI:** 10.3389/fvets.2026.1913012

**Published:** 2026-10-13

**Authors:** Julia Catharina Dose, Madlen Sarah Matz, Tiina Harmas, Marjo Hytönen, Emma Hakanen, Hannes Lohi, Thomas Parmentier, Holger Andreas Volk, Jasmin Nicole Nessler, Sofie F. M. Bhatti, Luisa De Risio, Paul Mandigers, Daisuke Ito, Fiona May Keir James, Tarja Susanna Jokinen, Sally Ricketts, Frank Steffen, Franziska Wielaender, Alexandra Eschenberg, Andrea Fischer, Andrea Tipold

**Affiliations:** 1 Department of Small Animal Medicine and Surgery, University of Veterinary Medicine Hannover, Hannover, Germany; 2 Small Animal Clinic, Centre for Clinical Veterinary Medicine, LMU Munich, Munich, Germany; 3 Department of Clinical and Medical Genetics, Faculty of Medicine, University of Helsinki, Helsinki, Finland; 4 Department of Veterinary Biosciences, Faculty of Veterinary Medicine, University of Helsinki, Helsinki, Finland; 5 Folkhälsan Research Center, Helsinki, Finland; 6 Faculty of Veterinary Medicine, University of Montreal, Montreal, QC, Canada; 7 Department of Small Animal, Faculty of Veterinary Medicine, Ghent University, Merelbeke-Melle, Belgium; 8 Mars Veterinary Health, Friars Gate, United Kingdom; 9 Department of Clinical Sciences, Faculty of Veterinary Medicine, University of Utrecht, Utrecht, Netherlands; 10 Department of Small Animal Clinical Sciences, Texas A&M University, College Station, TX, United States; 11 Department of Clinical Studies, Ontario Veterinary College, University of Guelph, Guelph, ON, Canada; 12 Department of Equine and Small Animal Medicine, University of Helsinki, Helsinki, Finland; 13 Canine Genetics Centre, Department of Veterinary Medicine, University of Cambridge, Cambridge, United Kingdom; 14 Department of Neurology, University of Zurich, Zürich, Switzerland; 15 Small Animal Clinic Oberhaching, Oberhaching, Germany; 16 Independent Researcher Hannover, Hannover, Germany

**Keywords:** breed, canine epilepsy, idiopathic epilepsy, phenotype, seizures

## Abstract

In response to the recurring identification of medication-resistant idiopathic epilepsy (IE) in English Springer Spaniels (ESS), the objective of this retrospective questionnaire-based observational study was to delineate the phenotypic characteristics of IE in this breed in comparison to a cognate breed, the English Cocker Spaniel (ECS), employing a novel, validated, owner-completed multilingual online questionnaire. Owners of 49 ESS and ECS with seizures completed the questionnaire, of which 16 ESS and 14 ECS fulfilled the inclusion criteria and were subsequently included in the study cohort, while the remaining questionnaires were excluded due to incomplete or inconsistent information or because the criteria for IE were not fulfilled. At the time of the survey 43.3% of the dogs had died, including five that died in association with epilepsy. The median age at seizure onset was 2.5 years in ESS and ECS (range: 0,5–9 years; IQR 2.0–4.0 years). Notably a high proportion of dogs in both breeds had experienced ≥1 cluster seizure and ≥1 status epilepticus. In ECS a significantly higher rate of marked reduction in seizure severity following initiation of anti-seizure therapy compared to the ESS (*p* = 0.009) was observed. Both breeds exhibit a moderate to severe disease course, with ESS showing a comparatively poorer response to antiseizure drugs. Furthermore, 43.3% of dogs with suspected IE also had affected relatives. The owner-reported occurrence of IE in related dogs may suggest a potential hereditary component in both breeds.

## 1 Introduction

Epilepsy is defined as the recurrent occurrence of focal and/ or generalized seizures, with clinical signs ranging from mild impairment of consciousness and minor motor disturbances to severe generalized tonic–clonic seizures (1, 2). An increase in neuronal excitability represents the common pathophysiological feature underlying all epileptic disorders (3). Epilepsy is suspected when two or more unprovoked seizures occur at an interval of more than 24 h apart (2) and is considered the most common chronic neurological disease in dogs (4) with reported prevalence ranging from 0.6 to 0.75% (5, 6). Prevalence is influenced by multiple factors (7, 8). An increased prevalence compared with the general canine population has already been identified in several dog breeds. Examples of breeds affected by IE include the Belgian Tervueren (7), the Labrador Retriever (8) and the Irish Wolfhound (9). In addition, male dogs are, on average, more commonly affected than females (5, 9, 10). According to the consensus statement of the International Veterinary Epilepsy Task Force (IVETF), seizures are classified by etiology into idiopathic epilepsy, structural epilepsy, and reactive seizures (3). Several studies have investigated breed-specific characteristics of idiopathic epilepsy to prepare genetic investigations. Severe disease courses and resistance to treatment with anti-seizure drugs have also been shown to occur more frequently in certain breeds, for example, in Border Collies and Australian Shepherds (11, 12). A severe clinical course was observed, characterized by frequently occurring cluster seizures, status epilepticus and drug resistance (11, 12). A study on idiopathic epilepsy (IE) in ESS has previously been conducted in a cohort from the United States (USA), indicating that ESS may respond inadequately to anti-seizure drugs and tend to develop a severe clinical course (13). The current study aims to determine whether these findings can be reproduced in an international, predominantly European cohort, thereby allowing broader generalization, and to use a previously validated questionnaire (14) for this purpose. The phenotype of IE in ESS was compared to the one of a related breed, the ECS.

## 2 Materials and methods

### 2.1 Questionnaire

This retrospective study is based on a questionnaire to study the phenotype of epilepsy and dyskinesia (14). The questionnaire was previously validated in a cohort of 606 dogs from 96 breeds and demonstrated and strong internal consistency and interrater agreement (14). Since 2021, the questionnaire has been available online and can be completed by dog owners via the following website: https://dogepilepsyresearch.org. Data included in the current study were collected between 2021 to April 2025. The novel questionnaire proved to be highly reliable during validation for consistently and comprehensively capturing various manifestations of epileptic seizures and other paroxysmal episodes across breeds, as well as for identifying breed-specific differences, thereby representing a robust tool for the description and characterization of epilepsy and movement disorders in different dog breeds (14). The aim of the questionnaire was to obtain as much information as possible about each dog’s seizure activity in order to characterize seizure semiology in detail and to support a reliable diagnosis of IE. Owners surveyed across five sections of the questionnaire addressing seizure type and characteristics, potential causes of seizures, medication, veterinary examinations and behavioral changes. The owners’ questionnaires were reviewed for plausibility, and to assess whether the reported cases were consistent with IE. All questionnaires were revied by one investigator (J.C.D). Unclear cases were discussed with a senior investigator (A.T.) and the finals classification was reached by consensus. General Data Protection Regulation (GDPR) (Art. 6(1)(e) in conjunction with Art. 89 GDPR, § 3(1) No. 1 NHG, § 13 NDSG) 89 were considered and processing was approved by the Data Protection Officers of the University of Veterinary Medicine Hannover, Small Animal Clinic LMU Munich, Department of Veterinary Biosciences University of Helsinki, Helsinki.

### 2.2 Study population

In total, the questionnaire was completed by 527 German-speaking owners, 841 Finnish-speaking owners and 390 English-speaking owners. Of these, 49 dogs belonged to the breeds English Springer Spaniel and English Cocker Spaniel. Four questionnaires were incomplete to an extent that precluded their inclusion in the analysis. Consequently, 45 questionnaires were assessed for the presence of suspected IE.

### 2.3 Recruitment

The study cohort was recruited through the databases of the University of Veterinary Medicine Hannover, the Ludwig Maximilians University of Munich, and the University of Helsinki, as well as through Kennel Clubs, announcements in a German print magazine for Springer Spaniel owners, and online forums.

### 2.4 Inclusion criteria

An overview of the inclusion and exclusion of dogs in the study is shown in Figure 1. Only questionnaires, in which all five sections were completed sufficiently to allow assessment of the seizure activity and evaluation of whether idiopathic epilepsy could be suspected, were included. Inclusion criteria for dogs with IE were based on the diagnostic criteria defined in the Consensus Statement on epilepsy diagnostics from the IVETF (3).

**Figure 1:**
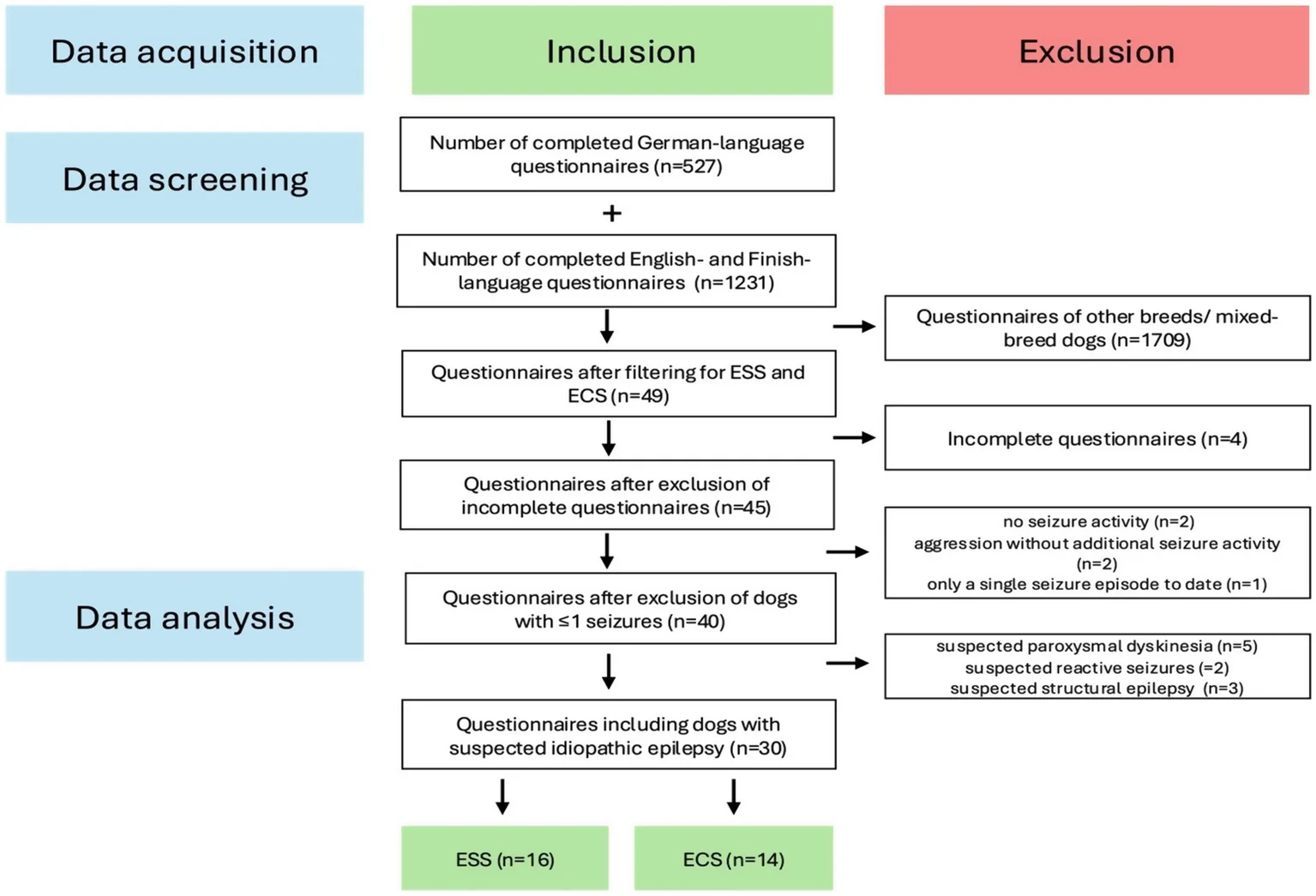
Flow-chart illustrating data acquisition, inclusion, exclusion and final study population (ESS: English Springer Spaniel; ECS: English Cocker Spaniel).

### 2.5 Exclusion criteria

Questionnaires that were incomplete to such an extent that no reliable diagnostic assessment was possible were excluded from the study. Dogs in which seizure-like aggressive behavior represented the episodic manifestation were excluded from the study due to the limited and inconclusive evidence available in the literature on this phenomenon (15–17). Dogs exhibiting exclusively focal motor episodes without impairment of consciousness or autonomic signs and with episode durations exceeding 5 min were classified as having paroxysmal dyskinesia (PD) (3), if no electroencephalographic (EEG) findings were available for the patient. The differentiation between epilepsy and PD was based on the IVETF-Consensus proposal: “Diagnostic approach to epilepsy in dogs” (3), a review on paroxysmal dyskinesia (18) and the International Veterinary Canine Dyskinesia Task Force ECVN consensus statement: Terminology and classification (19).

### 2.6 Statistical analysis

Survey data were prepared for statistical analysis using Microsoft® Excel (version 16.100.2). Statistical analysis was performed using SAS Studio 3.81 (SAS Institute Inc., Cary, NC, USA). The two breeds were analyzed both separately and jointly. For all categorical answers, the absolute frequencies were calculated; descriptive statistics were obtained for variables with nominal outcome measures. Graphical representations of the statistical results were generated using Microsoft® PowerPoint (version 16.100.2) and GraphPad Prism (version: 10.5.0 Graph Software, LLC, Boston, MA, USA). For metric variables, mean and median were calculated, and histogram inspection was used to assess normal distribution. Associations between qualitative variables were evaluated using logistic regression. Statistical significance was assessed using the Fisher’s exact test. A *p*-value of < 0.05 was considered statistically significant.

## 3 Results

### 3.1 Study population and diagnostic evaluation

A total of 30 dogs were included in the analysis (see Figure 1), comprising 16 ESS and 14 ECS. Sex distribution was as follows: 43.3% of the dogs were female (*n* = 13), of which five were neutered, while the remaining 56.7% were male (*n* = 17), including four neutered males. At the time the questionnaire was completed, 56.7% of the dogs were still alive (*n* = 17). Within the ESS group, 8 dogs had already died by the time of the survey. Five owners reported epilepsy as the cause of death (38.5%). All dogs were under veterinary care due to seizure activity (*n* = 30). Based on the veterinary diagnostic procedures performed, dogs in this study with suspected IE could be classified into confidence levels (TIER) according to the Consensus Statement: “Diagnostic approach to epilepsy in dogs” by the IVETF (3). Within the study population, 93.3% of the dogs (*n* = 28) had undergone diagnostic investigations sufficient to meet the criteria for diagnosis at TIER I. No dog fulfilled the criteria for diagnosis at TIER II. Although three dogs had undergone MRI and CSF examination, assessment of liver function by pre- and postprandial bile acid testing had not been performed. Two dogs (both ESS) met the criteria for diagnosis of IE at TIER III. In one additional dog (ESS), electroencephalography had been performed and pre- and postprandial bile acid testing was not conducted.

### 3.2 Seizure characteristics

Generalized epileptic seizures occurred in 87.5% of ESS (*n* = 14) and in 100% of ECS (*n* = 14), corresponding to 93.3% of dogs across breeds in this study (Table 1). In 50% of dogs exhibiting generalized seizures, seizures were reported to begin focally and generalized subsequently (*n* = 14; ESS: *n* = 8, ECS *n* = 6). In six dogs, no focal onset of generalized seizures was observed (ESS *n* = 4, ECS *n* = 2), while eight owners were unsure whether generalized seizures had ever started focally. All dogs exhibited autonomic signs during generalized seizures. Seizure frequency analysis showed that generalized seizures occurred monthly in 25% of the dogs and every 2–3 months in another 25% of the dogs (*n* = 7 each). In 50% of the dogs, the duration of generalized seizures ranged between 2 and 5 min according to the owners (*n* = 14). Binary logistic regression revealed no significant breed-associated difference in the duration of generalized seizures (*p* = 0.72). Focal seizures occurred in 60% of the dogs (*n* = 18), among these dogs, 10 were ESS and 8 were ECS; in each breed, two dogs experienced exclusively focal seizures without secondary generalization. Absence-like episodes were observed in 20% of the dogs (*n* = 6), with three dogs affected in each breed. In addition, 6.7% of owners reported other episodic events involving unusual movements or abnormal behaviors that could not be classified within the predefined seizure categories (*n* = 2; both ECS). Hypersalivation occurring without other signs of seizure activity and without any identifiable alternative medical explanation was reported in 13.3% of the dogs (*n* = 4; ESS: *n* = 3; ECS: *n* = 1).

**Table 1:** Seizure types observed in 30 dogs of the English Springer Spaniel (ESS: *n* = 16) and English Cocker Spaniel (ECS: *n* = 14). Values are presented as number of dogs (%). Percentages refer to the respective breed totals; multiple seizure types could be reported per dog.

Seizure type	All dogs (*n* = 30)	ESS (*n* = 16)	ECS (*n* = 14)
Generalized	28 (93.3%)	14 (87.5%)	14 (100.0%)
Focal	18 (60.0%)	10 (62.5%)	8 (57.1%)
Generalized seizures with focal onset	14 (46.7%)	8 (50.0%)	6 (42.9%)
Absence-like episodes	6 (20.0%)	3 (18.8%)	3 (21.4%)
Episodic events involving unusual movements or abnormal behaviors *	2 (6.7%)	/	2 (14.3%)
Episodes of hypersalivation*	4 (13.3%)	3 (18.8%)	1 (7.1%)

*Without other signs of a seizure.

Across both breeds and independent of seizure type, the median age at first seizure onset was 2.5 years (ESS range: 0.5–9 years; ECS range: 1–5 years; IQR 2.0–4.0 years).

### 3.3 Status epilepticus and cluster seizures

At least one episode of status epilepticus (SE) occurred in 30% of the dogs (*n* = 9), comprising 25% of ESS (*n* = 4) and 35.7% of ECS (*n* = 5). Cluster seizures (CS) had occurred at least once during the dog’s lifetime in 60% of cases (*n* = 19), affecting 56.3% of ESS (*n* = 9) and 64.3% of ECS (*n* = 9). Among dogs exhibiting CS of the generalized tonic–clonic seizure type, 62.5% consistently or almost consistently fully recovered between individual seizures (*n* = 10; ESS: *n* = 3, ECS: *n* = 7). Both, ≥1 status epilepticus and ≥1 cluster seizure were reported in 23.3% of dogs (*n* = 7), including 12.5% of ESS (*n* = 2) and 35.7% of ECS (*n* = 5). Either at least one episode of SE or at least one occurrence of CS was reported in 66.7% of dogs (*n* = 20), affecting 68.8% of ESS (*n* = 11) and 64.3% of ECS (*n* = 9). No significant difference was found between the two breeds regarding the frequency of SE (*p* = 0.69) or CS (*p* = 0.72) (Figure 2).

**Figure 2:**
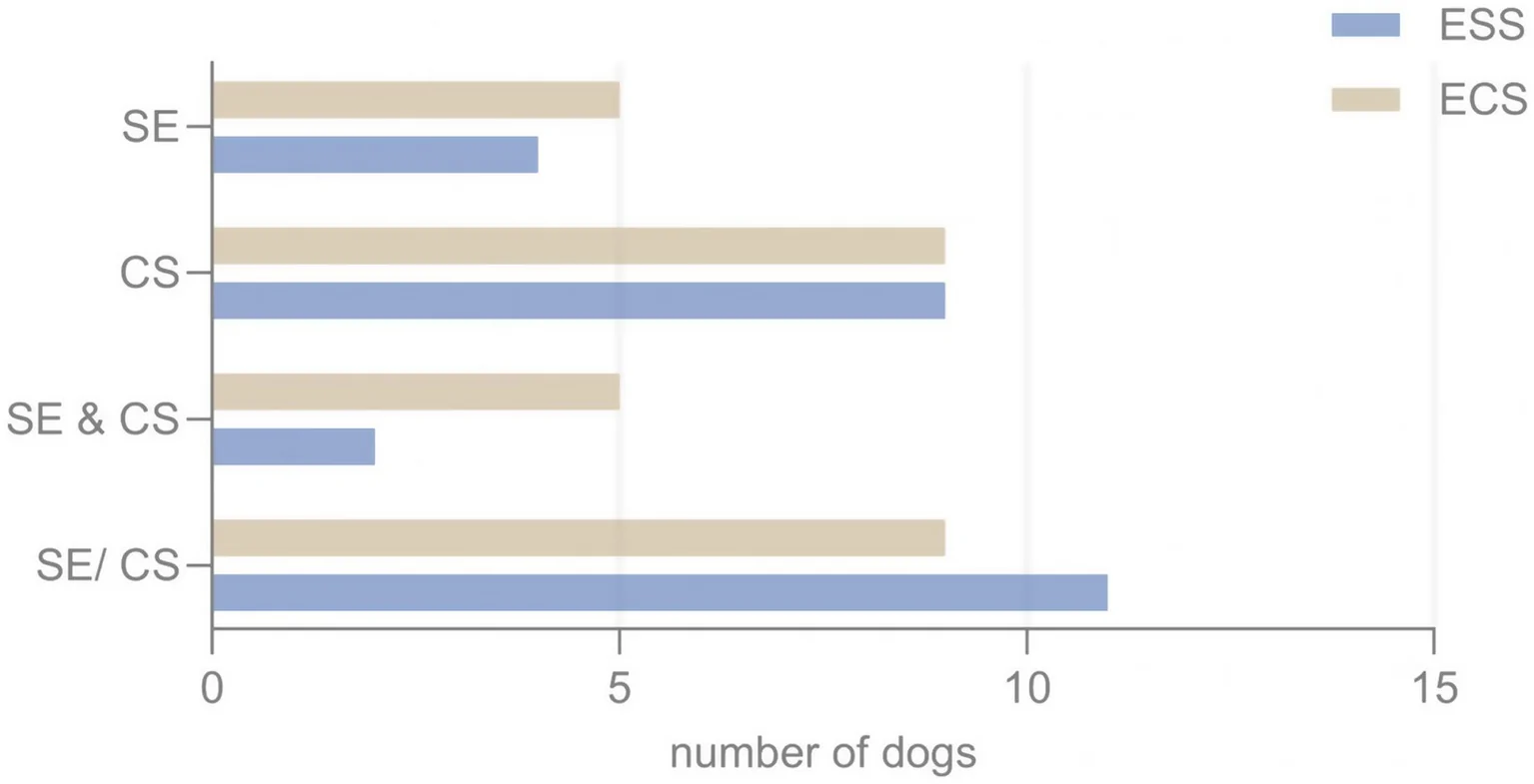
Number of dogs with a history of status epilepticus (SE), cluster seizure (CS), both SE and CS, or at least one of the two conditions (SE/CS) out of a total of 30 dogs, including 16 English Springer Spaniel (ESS) and 14 English Cocker Spaniel (ECS).

### 3.4 Pre- and postictal behavior changes

Behavioral changes occurring seconds to minutes before observation of generalized epileptic seizures were reported by 50% of owners (*n* = 14), including six ECS owners and eight ESS owners. Reported preictal behavior in the seconds or minutes before generalized epileptic seizures, listed in decreasing order of frequency, included staring into space (*n* = 7), restlessness (*n* = 5), vomiting (*n* = 3), attention-seeking behavior (*n* = 3), hypersalivation (*n* = 2), fearful behavior (*n* = 1) and other (*n* = 1). Of owners whose dogs exhibited generalized seizures, 78.6% reported postictal behavior changes (*n* = 22), 59.1% of dog owners indicated that normalization of behavior occurred within 30 min to 2 h. The most frequent reported postictal signs were disorientation (*n* = 17), aimless wandering (*n* = 16), unsteady gait (*n* = 15), transient blindness (*n* = 8), lethargy (*n* = 7), a strong urge to go outside (*n* = 7) and an increased desire to eat and drink (*n* = 7).

### 3.5 History

Occurrence of seizures in the family of the reported dog was highlighted by 43.3% of owners (*n* = 13). This included 43.8% of ESS (*n* = 7) and 42.9% of ECS (*n* = 6). At the time of questionnaire completion, 30% of dogs were neutered (*n* = 9), comprising 31.3% of ESS (*n* = 5) and 28.6% of ECS (*n* = 4). Of these dogs, 44.4% had been neutered prior to the onset of the first seizure event (*n* = 4). In none of the five dogs, that were neutered after their seizure onset did owners report a positive effect of neutering on seizure severity or seizure frequency.

### 3.6 Antiseizure drugs (ASDs)

Overall, 76.7% of dog owners reported that their dogs were receiving medication for seizure control at the time the questionnaire was completed (*n* = 23), comprising 62.5% of ESS (*n* = 10) and 92.9% of ECS (*n* = 13). Among the dogs for which the owners were able to provide information about the used ASDs (*n* = 20), 55% were managed with monotherapy (*n* = 11; ESS *n* = 4, ECS *n* = 7), receiving a single antiseizure drug, whereas the remaining 45% received polytherapy (*n* = 9; ESS *n* = 5, ECS *n* = 4), defined as treatment with two or more ASDs. 75% of dogs receiving ASDs were treated with phenobarbital (*n* = 15), making it the most commonly used medication in this study, followed by imepitoin (*n* = 6) and levetiracetam (*n* = 6). Only 17 owners were able to provide information regarding the effect of ASDs on their dog’s seizures frequency and severity. Seizure frequency: A marked reduction in seizure frequency was reported in 52.9% of dogs (*n* = 9), comprising 3 ESS and 6 ECS. Of these dogs, seven were treated with phenobarbital, four of them as monotherapy. A moderate reduction in seizure frequency was reported by 23.5% of owners, all of the dogs were ESS (*n* = 4). Even though the outcome was numerically more frequent in ESSs than in ECSs, the difference did not reach statistical significance (*p* = 0.08). Seizure severity: A subjectively moderate reduction in seizure severity since treatment initiation was reported by 23.5% of owners (*n* = 4), including three ESS and one ECS. A marked reduction in seizure severity was reported by 29.4% of owners (*n* = 5); all these dogs were ECS and exhibited a significantly higher rate of marked reduction in seizure severity compared to ESS (*p* = 0.009). Overall, 76.5% of dogs demonstrated treatment responsiveness in terms of a moderate or marked reduction in seizure frequency and/or severity (*n* = 13; ESS *n* = 7, ECS *n* = 6). No improvement in seizure frequency or severity was reported by 23.5% of owners. Independent of the specific medication used, 29.4% of owners whose dogs were receiving ASDs and who completed the questionnaire section on treatment effects reported that their dogs had been seizure-free since initiation of the respective medication (*n* = 5; ESS *n* = 1, ECS *n* = 4). No predefined minimum duration was specified for classification as seizure-free. Overall, of the dogs reported to be seizure-free at the time of the survey, four were receiving monotherapy and one was receiving polytherapy. All dogs receiving phenobarbital that were seizure-free at the time of the survey were ECS. Of the 23 dogs for which owners reported ASD administration, regardless of whether the specific medication was known, 39.1% were reported to exhibit adverse effects at the time of the survey (*n* = 9).

## 4 Discussion

This study aimed to characterize and compare the IE phenotype in ESS and ECS with a validated questionnaire. (14). While the age of seizure-onset and the seizure-characteristics were similar in the ESS and ECS and both breeds tend to show a moderate to severe disease course, the ECS appeared somewhat more prone to CS and SE, but without a statistical significance. Regarding treatment response, ECS demonstrated better responsiveness to ASDs compared to ESS, reflected in the significantly more frequent owner reports of a marked reduction in seizure severity. While sex distribution and age at seizure onset were consistent with the previous breed-specific study of the ESS (13) and cross-breed reports (20), including the slight male predisposition in the ESS and median onset around 2.5 years in both breeds, which is comparable to the Finnish Spitz (21), the Border Collie (22) and the Australian Shepherd (12). Seizure semiology and the proportion of generalized versus focal epileptic seizures were comparable to other published breed cohorts including the ESS study from Patterson et al. (13) and were also similar between the ESS and ECS in the current study. Focal-onset seizures with secondary generalization were observed in 50% of ESS and in 42.9% of ECS, which is comparable to those reported for the Spinone Italiano (23) and for the Petit Basset Griffon Vendéen (24), whereas other breeds exhibit even higher rates of seizures with secondary generalization (22, 25). Historically, focal seizures have been associated with intracranial lesions (26–28). Indeed, in a retrospective study, focal seizures were reported more frequently in dogs with “symptomatic epilepsy“, including reactive seizures and structural epilepsy (29). However, the IVETF Consensus Statement: “Diagnostic approach to epilepsy in dogs” explicitly states that seizure type alone should not be used to predict the underlying etiology (3). In a prospective study of 106 dogs, focal-onset seizures with secondary generalization were the most common seizure type in both idiopathic and structural epilepsy (20). Both breeds exhibited a moderate to severe disease course, primarily driven by the high proportion of dogs experiencing CS and SE. At least one SE occurred in 25% of ESS and 35.7% of ECS, while ≥1 CS was reported in 56.3 and 64.3%, respectively. Mild disease courses, characterized by isolated seizure events, are distinguished from moderate courses, defined by the occurrence of ≥1 CS and severe courses, characterized by the occurrence of ≥1 SE (11, 12). Approximately one quarter of the study population had experienced both CS and SE. Compared with other breed-specific studies describing moderate to severe forms of IE, e.g., the Australian Shepherd and the Great Swiss Mountain Dog (12, 25) both breeds in the present study can likewise be characterized as having a moderate to severe disease course. Although no statistically significant breed difference was identified, the numerically higher proportions of cluster seizures and status epilepticus in ECS suggest possible breed-related variability that requires investigation in larger cohorts. Given the established association of CS and particularly SE with poorer prognosis and reduced survival time in previous studies (30, 31), the high prevalence of these severe manifestations is clinically relevant for both breeds. Pre- and postictal behavioral changes were frequently reported in both breeds (ESS and ECS), especially postictal signs were observed in 71.4% of ECS and 85.7% of ESS. The most frequently reported postictal signs were disorientation, wandering, unsteady gait, transient blindness, lethargy, and a strong urge to go outside. In a cross-breed study, disorientation, aimless wandering, ataxia, and blindness were identified as the postictal signs most negatively affecting the quality of life of both dogs and their owners (32). Regarding therapeutic response, ECS showed a significantly higher rate of marked reduction in seizure severity after initiation of ASD treatment compared to ESS (*p* = 0.009). And 4 ECS were seizure-free after initiation of the respective medication compared to 1 ESS. While drug-resistance cannot be concluded given the lack of serum level data, dosing information and longitudinal follow-up, the comparatively poorer reported response in ESS is noteworthy and hypothesis-generating. A higher proportion of ESS received polytherapy, which may indirectly suggest more challenging seizure control, although this difference was not statistically significant. No significant association between the occurrence of CS/SE and reported treatment response was detected; however, the limited sample size and cross-sectional design likely reduced the ability to identify such relationships. Notably, a previous study has reported an association between the occurrence of CS and a poorer treatment response (33). In the present study, 39.1% of owners reported adverse effects in their dogs, regardless of the ASD administered. Among dogs receiving phenobarbital as monotherapy, 33.3% experienced side effects, compared with 25% of those treated with imepitoin. In the literature, imepitoin and levetiracetam are generally considered to be associated with fewer adverse effects than phenobarbital (34). Adverse effects may negatively influence the owner’s perception of their dog’s quality of life and may also impact the quality of life of the owners themselves (35). Therefore, when selecting ASDs, it is essential to adopt an individualized approach that aims to achieve the greatest possible reduction in seizure frequency and severity while minimizing adverse effects, thereby taking into account the quality of life of both dog and owner (36).

Nearly half of the dogs had affected relatives, supporting a probable hereditary component of IE in both breeds. In six dogs, multiple relatives were affected. In certain breeds, genetic loci associated with IE have already been identified, including regions containing or located near the ADAM23 gene (37). As the Fédération Cynologique Internationale (FCI) does not define minimum breeding ages, national breed organizations regulate breeding eligibility. In Germany, the “Verband für das deutsche Hundewesen (VDH) “permits breeding from 15 months in females and 12 months in males (38). Given that the median age at seizure onset in this study was 2.5 years in both breeds, affected dogs may already have been bred multiple times before clinical manifestation of the disease.

We recognize several limitations of this study, including considerations related to the use of the questionnaire in infrequently kept breeds. The small sample size of the two breeds represents a major limiting factor of this study. The strengths of the questionnaire lie in its comparability, scalability, and level of detail (14). However, to achieve these strengths the survey includes detailed questions and respondents need to watch video samples of seizures. Therefore, only two respondents completed the survey in less than the maximum recommended duration of 28 min identified in a study on optimal online survey length (39). The relative rarity of the breed - particularly in Germany – and the extensive nature of the questionnaire may have contributed to the limited sample size. Of all questionnaires available for analysis (*n* = 1.758), only 30 were completed by owners of ESS, meaning that 1.71% of responses were from the breed of interest and could be included in the initial analysis. In contrast, at the time of questionnaire validation by Matz et al. (14), 24.4% of dogs included were Labrador Retrievers. As this study was not designed as a prevalence study, or a formal feasibility or instrument-optimization study, no definitive conclusions can be made regarding the impact of questionnaire design on recruitment. Nevertheless, the respondent burden associated with obtaining detailed phenotypic information may have reduced owner participation, while breed rarity and the comparatively low prevalence of IE may have further limited the available study population. In addition, recruitment through university hospitals, breed clubs, breed magazines and online forums may have introduced selection bias, as owners of dogs with more severe or clinically recognized epilepsy may have been more likely to participate.

In addition to the retrospective cross-sectional nature of this study, which does not allow a more detailed assessment of seizure freedom or long-term response to ASDs, the questionnaire-based design may have introduced recall bias. Owners may not have accurately recalled seizure frequency, seizure severity or changes in these parameters following treatment. Treatment response was based on owner perception rather than standardized prospective seizure recording and should therefore be interpreted with caution. A further major limitation is the heterogeneous diagnostic certainty within the study population, as most dogs fulfilled only IVETF TIER I criteria, none fulfilled TIER II criteria and only two reached TIER III following the Consensus Statement of the IVETF (3). Therefore, alternative causes of seizures could not to be excluded with the same level of confidence in all dogs. Future studies combining detailed phenotypic characterization with prospective follow- up and genomic analyses may help to further elucidate breed- specific characteristics and the genetic background of IE in ESS und ECS. In addition, the relatively large number of statistical comparisons performed in this small study population may have increased the risk of type I error.

## 5 Conclusion

Idiopathic epilepsy in ESS and ECS showed a largely similar clinical phenotype, with comparable age at seizure onset, seizure type distribution, and a high proportion of dogs experiencing cluster seizures and status epilepticus, indicating a moderate to severe disease course in both breeds. Owner-reported treatment response suggested a greater reduction in seizure severity in ECS than in ESS, although this finding should be interpreted cautiously because of the limited sample size and lack of standardized longitudinal treatment data. The frequent reports of affected relatives support a suspected hereditary basis for IE in both breeds. Overall, these findings expand the clinical characterization of IE in two infrequently studied breeds and indicate that the questionnaire is suitable for detailed phenotyping.

## Data Availability

The original contributions presented in the study are included in the article, further inquiries can be directed to the corresponding author/s.
